# Feasibility of digital footprint data for health analytics and services: an explorative pilot study

**DOI:** 10.1186/s12911-016-0378-0

**Published:** 2016-11-09

**Authors:** Marja Harjumaa, Saila Saraniemi, Saara Pekkarinen, Minna Lappi, Heidi Similä, Minna Isomursu

**Affiliations:** 1 VTT Technical Research Centre of Finland, P.O.BOX 1100, 90571 Oulu, Finland; 2 Oulu Business School, University of Oulu, P.O. BOX 4600, 90014 Oulu, Finland; 3 Information Technology and Electrical Engineering, University of Oulu, P.O. BOX 3000, 90014 Oulu, Finland

**Keywords:** Digital footprint, eHealth, My data, Qualitative inquiry, Expectations, Experiences, Self-care

## Abstract

**Background:**

As a result of digitalization, data is available about almost every aspect of our lives. Personal data collected by individuals themselves or stored by organizations interacting with people is known as a digital footprint. The purpose of this study was to identify prerequisites for collecting and using digital data that could be valuable for health data analytics and new health services.

**Methods:**

Researchers and their contacts involved in a nationwide research project focusing on digital health in Finland were asked to participate in a pilot study on collecting their own personal data from various organizations of their own choice, such as retail chains, banks, insurance companies, and healthcare providers. After the pilot, a qualitative inquiry was adopted to collect semi-structured interview data from twelve active participants in the pilot. Interviews comprised themes such as the experiences of collecting personal data, as well as the usefulness of the data in general and for the participants themselves. Interview data was then analyzed thematically.

**Results:**

Even if the participants had an academic background and were highly motivated to collect and use their data, they faced many challenges, such as quite long delays in the provision of the data, and the unresponsiveness of some organizations. Regarding the usefulness of the acquired personal data, our results show that participants had high expectations, but they were disappointed with the small amount of data and its irrelevant content. For the most part, the data was not in a format that would be useful for health data analytics and new health services. Participants also found that there were actual mistakes in their health data reports.

**Conclusions:**

The study revealed that collecting and using digital footprint data, even by knowledgeable individuals, is not an easy task. As the usefulness of the acquired personal health data mainly depended on its form and usability for services or solutions relevant to an individual, rather than on the data being valuable as such, more emphasis should be placed on providing the data in a reusable form.

**Electronic supplementary material:**

The online version of this article (doi:10.1186/s12911-016-0378-0) contains supplementary material, which is available to authorized users.

## Background

As a result of digitalization, data is now available about almost every aspect of our lives. Individuals are developing an ever-increasing personal *digital footprint* by using different information and communication technology (ICT) solutions and services from public and private organizations. As a consequence, large amounts of data are stored within the databases, which individuals manage by themselves, but also within the databases of different organizations [1]. Digital footprints are sources of information about the behavior of individuals, as well as groups of people [2, 3].

There are visions that personal digital footprints could be used to improve people’s health and wellbeing, especially in managing lifestyle-related diseases and their risks (e.g. [4, 5]). Digital solutions could help users to engage with new, healthier behaviors by being constantly present in their everyday lives and providing useful content and functions [6]. The process of using data has many stages, starting from the preparation and collection of data, then continuing to the integration of different data sources, reflecting on the data, and finally using the data [7]. Studies have shown that there are several barriers that prevent individuals from collecting data, such as lack of time, difficulties in collecting data from multiple inputs, and understanding the format of the data collected [7].

Although using the data collected through personal devices and applications has been studied before, as well as patients’ engagement with personal health records (e.g. [8]), less is known about the use of data stored in the databases of public and private organizations. These data sources include many sources that are not traditionally considered to be “health data”, but that can clearly be useful in maintaining a healthy lifestyle. As an example, consumption data collected by retail chains or mobility data collected by public transportation companies are such useful information that people might be interested. There is no doubt that individuals could also benefit from services based on more exact knowledge about their everyday lives [9]. This knowledge could, for example, provide support and motivation for individuals, as well as a more thorough understanding of their condition for healthcare professionals.

There is not much information on how people experience collecting their digital footprint and how they use the data they receive from the databases managed by different organizations. This study will contribute to the research by exploring the feasibility of digital footprint data stored by organizations with which individuals interact in everyday life. The research question is as follows:

1. How feasible it is to collect digital footprint data stored by organizations and use it in health data analytics and health services?

We approach the research question by exploring the experiences of a group of researchers and their contacts on the data collection process and on their perceived usefulness of the data. People’s expectations of how the digital footprint could be used in the future are also analyzed. Although the participants are more knowledgeable in digital health applications and devices than people on average, we expect that the study will give new insight for planning and conducting similar studies with other target groups.

As our aim is to capture the dynamic nature of individual experiences in collecting, using, and sharing the digital footprint data, we follow the approach of Tronvoll et al. [10], who suggest that research should involve the behavioral part of activities, interactions, experiences, processes, and relationships, and should be conducted together with the research object. In adopting this approach, researchers and participants are interactively linked and have influence on each other, gradually developing a more complete understanding of the phenomenon [11]. Following Kuzel ([12], p. 34), this inquiry does not aim to generalize or predict, but rather to create and test interpretations.

This paper extends the work of Gencoglu et al. [13], where the overall success rate of the information requests and the format of the received data were studied and reported. In their study, 11 active participants sent 100 information requests during a period of five months. The percentage of requests answered (i.e. the response rate) was 75.0 % and the percentage of requests answered with data or instructions on how to reach the data (i.e. the data reception rate) was 61.0 %. Both measures varied between 15 data source categories, such as banking, groceries, and healthcare. In the healthcare category, the response rate was 76.7 % and the data reception rate was 56.7 % [13]. The data in this study comprises a sample in which participants were chosen based on their involvement in the above-mentioned study. The following section will describe the method and research process in more detail.

## Methods

### Study design

#### Pilot setting

This study examines the experiences of a group of researchers and their contacts in collecting their own digital footprints. Originally, 20 participants involved in nationwide research projects focusing on digital health in Finland were asked to participate in a pilot study of collecting their own personal data from various organizations of their own choice. The participants were instructed to send out data requests to organizations, to collect their digital footprint data. They could also use a list of potential target organizations, which were defined in a collaborative effort by the multidisciplinary researcher team involved in the research design. Organizations included retail chains, banks, electricity companies, insurance companies, telecommunication service providers, the national archive of health information, hospital districts, health centers, private healthcare providers, and fitness centers. Participants were asked to consider data sources that could be useful for understanding their own health and health behavior, current or past. The data request form and cover letter were prepared for requesting the data to be delivered to the participant’s email or home address. The request form preferred data delivery over API, or on a memory stick or DVD, rather than printed paper documents. Participants were also asked to collect their health data themselves, using self-monitoring devices and applications, such as Withings Pulse and technologies they already owned, but not all participants took part in this self-monitoring. Thus, the results are not presented in this article.

The participants signed an informed consent form prior to the study, and were aware of their right to withdraw from the study at any time. In addition, the participants were instructed to go through the data they received, and to remove data they considered to be sensitive personal information, when necessary, prior to handing it over for research purposes (see [13]). Ethics committee approval was not acquired, as the participants were not exposed to any intervention or treatment, and the focus was mainly on studying the data request process and their personal experiences.

In Finland, legislation allows individuals the right to see their personal data stored by organizations. Therefore, it was expected that all organizations would be prepared to reply to the data requests sent by participants, and to provide a copy of their personal data. Gencoglu et al. [13] have already reported quantitatively the results of a data request sample, as described above in the Introduction.

#### Qualitative inquiry

After the data request pilot, to achieve a more in-depth understanding of the experiences, a qualitative inquiry was adopted to collect semi-structured interview data from active participants [14]. Participants were considered to be active if they provided their data for the research program. In the pilot study, eleven participants provided their data, but after its analysis, one more participant provided her data. Thus, twelve participants were chosen for qualitative interviews. Eight of them were women and four were men. They were aged from 24 to 49 years. All the participants had an academic background, which varied from computer engineering to business and economics. Most participants had previous experience of digital health applications and devices, either from a user’s perspective or from a research perspective. Thus, their experiences represent more innovators’ and early adopters’ experiences than experiences of the general public using services on a regular basis. Empirical data for this qualitative inquiry was collected by Skype or telephone, or through face-to-face contacts [Table 1]. The interviewees were encouraged to speak freely and to provide information that they themselves considered relevant ([15], p. 48), but framed by the interview guide. Interviews comprised themes such as experiences of collecting personal data, meaning the data request process, as well as the perceived usefulness of the data for themselves and in general. In addition, interviewees were also urged to think about their attitudes towards the data: their willingness to share it for different purposes, such as preventive health and wellbeing services, and their willingness to take an active role in managing access rights to it. Interviewees were also asked about their earlier experiences of collecting personal data and using it for maintaining a healthy lifestyle [see Additional file 1].

**Table 1:** Interview protocol for four male and eight female participants

ID	Participant’s gender	Interviewer	Analyzer
#1	Female	1. author	1. author
#2	Male	1. author	1. author
#3	Female	1. author	1. author
#4	Female	1. author	2. author
#5	Female	1. author	2. author
#6	Male	2. author	2. author
#7	Female	3. author	4. author
#8	Female	3. author	3. author
#9	Male	3. author	1., 2., and 3. authors
#10	Male	3. author	4. author
#11	Female	3. author	4. author
#12	Female	3. author	2. and 3. authors

Interviews were conducted in June 2015, using a guide to interview topics, by three researchers (the 1^st^, 2^nd^, and 3^rd^ authors of this article), who did not participate in the study as research subjects. Interviews were recorded (with consent) and transcribed verbatim, producing 109 pages for the analysis. Notes were made at the time of the interview. Interviews lasted approximately 60 min.

### Data analysis

Health and wellness data is shaped by expectations of how the data is collected, stored, used, and shared, as influenced by laws, regulations, norms, and values [16]. Evaluating subjective experiences is tricky, as the experience of one person cannot be directly observed, captured, or analyzed by another person [17]. Cognitive scientists have recognized the challenges of reliability of first-person reports about subjective experience, and the danger of changing those experiences by asking people to describe and recollect past experiences [18].

In this study, we rely on narrative first-person descriptions of subjective experience, supported by the logged data collection history of individuals [15, 19]. Thus, recollections of experiences are evoked with the help of actual data records received by participants, and the log history describing where they sent data requests and how companies responded.

Transcripts were analyzed thematically in the light of the themes in the interview guide [20]. Data analysis followed a process, in which at first those researchers who conducted the interviews read the transcripts carefully and systematically identified themes and subthemes that were associated with the research questions. Two interviews were analyzed by more than one researcher, in order to confirm a shared understanding of the data. The data was then organized in a text document according to the themes. The researchers then had two analysis sessions in which they shared their preliminary thoughts and findings, and discussed the thematic framework. In the second phase, three other researchers joined the team and familiarized themselves with the analysis document. According to this, the thematic framework was further refined. Finally, the data was interpreted and findings were reported as an outcome of a joint analysis process.

The analysis focused on the *subjective experience* of the interviewees as participants in the data request pilot study [13]. The analysis concentrated on (1) the experiences of the participants during the data request process, meaning how they experienced the interaction with the organizations to which they sent their data requests, and (2) the experience concerning the perceived usefulness of the data, meaning whether the participants found the data to be valuable and useful for them. Within an organizational context, perceived usefulness has been defined as “the degree to which a person believes that using a particular system would enhance his or her job performance” [21]. We define perceived usefulness as a parameter describing the extent to which the subjects believe the data will help them in their activities related to achieving better health. In other words, we explore value from the individual’s perspective [22], that is, how the participants talked about the data [16].

## Results

### Experiences of collecting and using the data

This section describes the main findings related to interviewees’ experiences of the data request process and using the data. In the following, the findings are grouped as follows: data request, form of data, and content of data.

#### Data request

Interviewees valued the background information to the pilot that was offered to them beforehand. The researchers developed a specific form for the data request process, making the process easier to manage for participants. However, some of the organizations from which data was requested had their own data request forms that interviewees needed to fill out, in addition to the pilot study’s own form, which caused extra work. Participants experienced that it was quite easy to send the data request, but most participants experienced some kind of challenge with the process later on.

Because organizations, especially in the health sector, had several registers, interviewees faced difficulties in finding the “right register” to direct the data request to. When they sent the data request, they did not get data from all registers at once. Interviewees had expected that the data would arrive considerably fast, but there was a quite a long delay in the provision of the data. The typical response time was approximately three months [13].

Not all organizations even reacted to the data requests, which was disappointing for the participants, because they would have expected at least a reply. In addition, the process did not always go as planned. One interviewee announced that a bank had tried to sell him the requested personal data. Several interviewees experienced that the data was not complete and that there was more information about them somewhere, but they were not given access to it. As one interviewee described her feelings:

*Well, I feel that I didn’t get the information they have, I’m sure they didn’t give it all. (#3)*

The way in which data requests are currently handled in most organizations was seen as old-fashioned by participants, and they considered that organizations “do not encourage” individuals to request and use their data.

#### Form of data

The data was often provided in written paper or PDF format, which was disappointing for many participants, as that hinders them from easily using the data for further analysis. A lot of time also had to be spent on checking all the information:

*…many provided the data in PDF format. It causes extra work. At least if I had wanted to upload the data into a spreadsheet format in order to do something. (#3)*

Participants also expected that if making data requests becomes more common, other people would also be disappointed:

*It might be, similar to my experiences, that it would be a disappointment that it (the received data) is in such a format that you can’t utilize it. (#3)*

Some organizations provided access to the data through a web interface, but the interviewees were not able to download the data for further use. They experienced that this data request process did not necessarily bring superior value compared to existing methods of providing access to use their own data, such as the current web services that organizations had (i.e. for members of retail membership programs).

#### Content of data

Mostly, the data received differed from what the respondents had expected. It was mostly registry information, which the participants did not find that interesting.

*I only got information about some current and past contracts, terms of agreement documents, something like this. I didn’t get any real information. (#1)*

*From many places I only got register information, it was not anything valuable as such, which I would have wanted to know, which they surely have. (#3)*

The participants also experienced that the data received from different organizations varied in quality:

*Well, let’s say that the quality varies a lot. As a whole that does not give a very coherent image. (#2)*

Interviewees’ positive experiences were mainly related to the information provided by healthcare providers. They considered it useful, because they expected to use it in the future by sharing it with their health-service providers. The content of the health reports was considered to be personal, interesting, complete, and even fun sometimes. As participants described their feelings:

*In my opinion, the only useful information came from private healthcare organization (anonymized). I got all information on treatments and doctors’ medical reports. (#1)*

*All data I have from different health services is useful and I can put it forward to my next doctor. (#9)*

*Health information, I probably wasn’t even aware of what people have written about me, what doctors have written and nurses at the maternity clinic, it was fun. (#3)*

Interviewees were surprised about the content, namely what information was provided to them, in how much detail, and how old some of the information that is stored can be. Interviewees stated that the experience was an eye-opener:

*If you now think about it rationally, you understand that companies store that kind of information, but in a way, eyes were opened with regards to how much information is stored there about me. (#1)*

Even though interviewees in this study were likely to be more familiar with personal data than individuals on average, because of their background in ICT or services research, they were surprised how much data was available in different databases. They also became more aware of the fact that personal data is valuable not only for themselves, but also for other actors, such as companies in the healthcare sector:

*I think that everyone should be aware of the fact that your data is valuable. Information is cash nowadays. (#6)*

Many respondents in interviews considered themselves to be early adopters of technology. Interviewees realized that health services based on personal data are still in the early phases of development. At present, participants had no high expectations concerning these services, but rather they thought that digital health-service development will be needed. Early adopters and groups such as Quantified-Selfers are genuinely interested in measuring themselves, and they are actually needed to increase general awareness of personal data, and health-related services based on personal data.

*If nobody talks about these, if there isn’t a living example of a person who actually does this, then people won’t become aware of the possibilities in the same way. (#7)*

*It is not about that, that there would be a certain group of people who are willing to try all kinds of things, but instead, there should be enough good services that attract people. (#10)*

### Expectations for the future use of data

Interviewees were asked to describe their expectations for the future use of data (c.f. 15). These expectations are subjective interpretations by interviewees of how the digital footprint data that they collected could be used in future services.

Although the participants experienced that, in its current form and with its current content, the data was not that valuable as such, they described their expectations for future footprint data.

*It just depends on what kinds of services innovative companies develop for these people. The data itself means nothing. But what will be developed based on that data. (#10)*

*all these kinds of data sources; we are not that good at observing our own behavior always, often decisions and things like that are made unconsciously and you are not always conscious of what you are doing or why and what the reasons are… (#2)*

Based on our interviews, in the future, the use of personal data should empower individuals to understand their health better and to support and enable self-care. In this study, self-care relates to the ability to manage one’s own life, especially related to health and wellbeing [cf. 23]. Participants expected that if health data was available for services, it could provide a more holistic view of an individual’s own life through, for example, the analysis and visualization of personal data.

*Yes, of course I monitor my sleep quite a lot. I’m more interested about the quality of sleep than how active I am. I would like to understand in a more detailed level why the amount of deep sleep has decreased and why I’m waking up or something like that. (#2)*

*Yes, it would reveal my own misunderstandings and illusions. It would probably give a more realistic view of my health status and would definitely be useful. (#9)*

Interviewees emphasized that organizations could better utilize and refine data to develop future self-care services. For example, these services could provide support for lifestyle changes, enabling people to be more responsible for their own health and wellbeing. To develop self-care services, the following aspects of personal data were identified through our analysis: visualizing data, sharing data, and integrating data sources.

#### Visualizing data

Participants expected that personal health data could be used for visualizing how everyday actions affect their own health and wellbeing, and how to avoid negative effects on their health:

*I still emphasize the visual aspects, you would not have to read tons of pages, but you could understand at one glance. You could make a visualization of your different treatments and when you have been vaccinated, as an example. (#4)*

#### Sharing data

Some of the interviewees would be willing to share their personal data, if this would enable the development of services of better quality for them. However, other interviewees had some severe worries about data sharing. Especially when it is a question of sensitive personal data, interviewees emphasized that they would share it only with organizations they could trust. Interviewees most trusted public actors, and especially healthcare organizations, with whom they were willing to share data. They also had positive attitudes toward sharing data for research purposes, which was quite understandable, as many of the participants were researchers.

*Yes, I would be ready to share my data between health organizations, I don’t see any reason why my health information from a hospital district (anonymized) couldn’t be available in a private health care organization (anonymized). So, if I have to trust somebody, I think that those (health organizations) are the ones that I have to trust. (#12)*

*Well, if you think about it, that as a consequence of “my data approach” a person could then share his information for research purposes and get feedback, I think that is a clear advantage. (#1)*

However, some interviewees were very skeptical about sharing their personal data with, for example, insurance companies and start-ups in foreign countries:

*This kind of a typical example, which is probably against the law also, is one’s own genes and insurance companies, although I doubt that there’s anything I would worry about, but intuitively I wouldn’t make a linkage between this kind of information. (#2)*

*Or if we are talking about an insurance company, I’m not sure what I would like to share. If they raise prices based on if they see how healthily I’m eating or if it influences something. (#3)*

*But I’m not ready to publish my own health data on the internet, for instance, because I’m afraid of insurance companies. (#9)*

Participants were worried about privacy risks related to data sharing and especially to cloud services. Some were even so worried that they would prefer local databases that are disconnected from the internet, to ensure data privacy.

Those who were willing to consider sharing data emphasized that control over their own data was crucial. Even though interviewees were mostly willing to share their data, possible benefits related to data sharing were difficult to see. It was important for the participants to be able to decide what kind of data they would share and with whom. For example, participants expected that they could share fitness data.

#### Integrating data sources

Integrated data sources played a significant role for interviewees. In addition, personal data integration requires sharing data with external actors. According to participants, integrating data from different sources automatically produces more reliable data, which, in turn, could provide better support for their health and wellbeing. Having their own data in one place could also increase the sense of control over their data:

*It would be good to have a single platform for those (consumers), so you could have that (data) through the internet. You could go there for a couple of times at most and you would have the data by yourself or you have said who is allowed to use it. (#7)*

### Summary of the results

Participants’ experiences revealed that many of the organizations with which people interact in their everyday lives do not currently have systematic processes to provide data. On the other hand, the data that organizations are willing to share under the current legislation was not perceived to be very useful by the participants in this study, because the content was not found to be relevant enough and the data was not in a reusable form. The most interesting data was received from healthcare providers, but the participants could only take an overview of it. The participants expected that, in order for the data to be useful for them, it should be possible to integrate data from different sources, visualize it, and share it with reliable partners.

## Discussion

The objective of this study was to find out how feasible it is to collect digital footprint data stored by organizations. The research question was approached through the experiences of a group of researchers and their contacts who were willing to go through the data collection process, donate their data for research purposes, and share their experiences of the process. The findings show that, currently, it is not very feasible to collect digital footprint data and its content is not what people expect.

While earlier studies have demonstrated that there are several challenges in using and reflecting on personally collected data [7], this study brought more understanding by exploring the practical conditions of using data within the databases of public and private organizations. Although the legislation provides individuals with a right to see their personal data, the study showed that, currently, most organizations are not prepared to provide data for individuals in a format that would be useful and practical from the viewpoint of health data analytics and new health services.

The findings suggest that the value of the acquired personal health data was mainly dependent on how the data could be utilized for services or relevant solutions, rather than the data being valuable as such. In order to manage their health, people need new support services for collecting, integrating, and reflecting on their own health data. In order to do this, the data needs to be in a reusable form and it needs to be compatible with the services available. Analysis of the experiences showed that individuals expect that the data could provide a more holistic view of their quality of life and the need for the change, through analysis or visualization of personal data. Interestingly, in earlier research, social and relational value has been largely identified (e.g. [20]), but it was not clearly present in this study. Instead, participants emphasized the meaning of integration of different types and sources of data in value creation, providing a more reliable and realistic view of their health and well-being.

Although the participants were more knowledgeable about digital health applications and devices than people on average, new insight was gained for planning and conducting similar studies with other target groups. To guide similar research settings, this study shows that it is necessary to allow individuals to go through the data they receive and remove data they consider sensitive before they hand it over for research purposes, because the content can be very different from what they expect. The study also points out that when several individuals have a need to request data at the same time, it is beneficial to contact key organizations together, in a centralized way. This allows the organizations to prepare in advance to respond to the data requests. In our research setting, these key organizations included dominant retail chains storing consumer data, as an example.

There are many possibilities for future research in this area. It would be interesting to expand this study to the general public, and also to explore the organization’s point of view. It is clear that the provision of data has financial costs. However, investing in ways of providing better access to personal data could open up opportunities for creating new services. Still, it is a risk for a company, because people are not yet used to using their data, and as our study showed, they do not necessarily know what information is collected about them. Thus, the first reaction of the general public can also be negative.

Besides financial issues, there are also ethical issues to be solved. As digitalization goes further, almost every aspect of people’s lives can be tracked somehow. People have different kinds of motivations and abilities to understand their data, especially health data. Currently, people can access some of their information, such as laboratory results and medicine prescriptions, but, for example, diagnoses are usually communicated directly by healthcare professionals. They can evaluate how much information the patient is capable of receiving, and the patient has the opportunity to ask questions instead of searching for mixed information online. Our results show that people can also be surprised about the content of their health reports.

One of the urgent challenges that both research and practice are trying to solve is creating value from data. This study explored the possibilities of individuals to collect their data from various organizations and to use the data in a meaningful way. The findings suggest that, before value can be created from data, it is necessary to develop technical solutions further (e.g. solutions that enable the integration of data from different sources) and also to create new services that use this data. In addition, as several participants noted that, in the future, data could be useful for empowering individuals to manage their health better and use self-care services, it is also necessary to increase the willingness of individuals to maintain a healthier lifestyle, and to make them aware of the possibilities of digital footprint data.

It has been acknowledged that lifestyle is the single greatest opportunity to improve health and reduce premature deaths, whereas medical care plays a relatively small role [23]. Furthermore, in the future, people can have a bigger role in their medical care: patients will be likely to prepare themselves in advance of consultations, communicate effectively with clinicians, and organize and store information for future use. Patients are expected to participate proactively, coordinating and managing their care among multiple stakeholders, as well as to interact and share health information [24]. This means that, in the future, individuals’ responsibilities and possibilities for managing their health are likely to increase. Digital footprint data has a lot of potential, but the tools and services needs to be developed further.

## Conclusions

This qualitative inquiry increased understanding of data collection and utilization related to using digital footprint data for health data analytics and new health services. The study setting is quite unique – a group of voluntary participants were willing to request their data, donate it to the research project, and then share their experiences on the process and their further use of the data. The study showed that organizations are generally not well prepared to provide the data, and in most cases it is not provided in a reusable format. Individuals also questioned the value and meaningfulness of the received data as such. The findings suggest that there is a need for new services that enable individuals to collect, integrate, and reflect on health data. This presumes that individuals have access to data within the databases of public and private organizations, and that it is in a reusable form.

## Additional file

Additional file 1: Interview guide. A while ago you were involved in so called minipilot, where you gathered your personal information from different sources of information and possibly used some device to gather personal data of yourself. (DOCX 17 kb)

## Abbreviations

API: Application programming interface
DVD: Digital video disc
ICT: Information and communication technology
