# Supplementary material for: Feasibility of digital footprint data for health analytics and services: an explorative pilot study
Source: BMC Med Inform Decis Mak. 2016 Nov 9;16:139. doi: 10.1186/s12911-016-0378-0 (PMC5112682; doi:10.1186/s12911-016-0378-0)
Supplement: Additional file 1: — Interview guide. A while ago you were involved in so called minipilot, where you gathered your personal information from different sources of information and possibly used some device to gather personal data of yourself. (DOCX 17 kb) [file 12911_2016_378_MOESM1_ESM.docx]

**Additional file 1 – Interview guide**

**A while ago you were involved in so called minipilot, where you gathered your personal information from different sources of information and possibly used some device to gather personal data of yourself.**

**Experiences**

Could you describe first your thoughts of this pilot?

If you could now decide, would you still participate in a similar pilot? Why / Why not?

What made you stay in the pilot?

You got to decide from which companies /organizations you asked data concerning yourself. What were those companies/organizations? Why did you choose particularly those sources of information?

What kind of experience was it to ask for information? Could you describe your experience from different organizations? E.g.

- What kind of information you asked from organizations?
- How did it succeed?
- What kind of challenges you faced?
- Did you get information you asked? What didn’t you get?
- Did the data you received correspond to your expectations? Did something surprise you? Why?
- Do you think this information is useful to you?

**Data use**

What kind of personal data you have gathered before this research?

- Consumption
- Body function
- Physical activity
- Symptoms
- Space and time
- Physiological variables
- Mental wellbeing
- Usage of time
- Anything else, such as usage of money, shoppings, social networks, learning results, transport and energy consumption

Where and how you have used the data you gathered?

How you familiarized yourself with the data you gathered from yourself in this pilot? How did you utilize this information?

What kind of feature or service would have helped you to utilize more this information?

What kind of information made you most enthusiastic? What kind of information felt useless?

What kind of data and in which form (from these sources of information) would be interesting?

What would you think if a certain source of information would offer you services based on your earlier buying behaviour or by combining information from different services/information sources?

If a certain source of information would offer services that would help you to see how your health is going to develop during years, would you use that kind of service? What kind of services those could be?

Did you utilize some device to gather your personal data during the pilot - what?

What kind of experience this was? What were pros and cons in this experience?

Did you utilize the data you gathered somehow? What would have you changed concerning the device / application?

What kind of thoughts gathering your personal data and getting to know your data caused?

**MyData and data sharing**

How your participation in this pilot has changed your perspective or way of thinking concerning myData / personal data and services based on that?

Now that you have seen what kind of information of yourself is available in different sources of information, how valuable do you experience your personal information?

Would you be ready to pay for services that would be built around your personal information, for example, personalized services? How much you would be ready to pay for these services? Could you give an example concerning a certain source of information in this pilot?

How willing would you be to utilize your data in form of services?

Would it made difference to how valuable you would experience your personal data, if you would gather information actively yourself, for example, through a certain smart device, or if your personal information would accumulate to a certain source of information, for example, through discount cards?

What kind of information you gathered would you be ready to give for companies to use? What wouldn’t you share? Why?

For which kind of organizations or companies would you be ready to share your personal health data?

Would you like to manage your personal digital data mass or would you like to outsource it to some external party (same way as people outsource investment portfolio management to their bank)?

**Final questions**

If you think your own experience through the eyeglasses of a researcher, what are the central benefits that an ordinary consumer can achieve through myData approach? What about disadvantages?

Finally, how would you describe a forerunner of myData usage? What kind person this kind of forerunner would be? How would this forerunner use myData services and how would he or she differ from other people?

Do you want to give feedback concerning this interview or would you like to comment something concerning the pilot?
